# Genetic trend for growth and wool performance in a closed flock of Bharat Merino sheep at sub temperate region of Kodai hills, Tamil Nadu

**DOI:** 10.14202/vetworld.2016.276-280

**Published:** 2016-03-16

**Authors:** P. K. Mallick, S. M. K. Thirumaran, R. Pourouchottamane, S. Rajapandi, R. Venkataramanan, G. Nagarajan, G. Murali, A. S. Rajendiran

**Affiliations:** 1 Southern Regional Research Centre, ICAR-Central Sheep and Wool Research Institute, Mannavanur, Kodaikanal, Tamil Nadu, India; 2 Post Graduate Research Institute in Animal Sciences, Kattupakkam, Kancheepuram, Tamil Nadu, India

**Keywords:** Bharat Merino sheep, breeding value, genetic trend, regression

## Abstract

**Aim:**

The study was conducted at Southern Regional Research Center, ICAR-Central Sheep and Wool Research Institute (CSWRI), Mannavanur, Kodaikanal, Tamil Nadu to estimate genetic trends for birth weight (BWT), weaning weight (3WT), 6 months weight (6WT), and greasy fleece weight (GFY) in a Bharat Merino (BM) flock, where selection was practiced for 6WT and GFY.

**Materials and Methods:**

The data for this study represents a total of 1652 BM lambs; progeny of 144 sires spread over 15 years starting from 2000 to 2014, obtained from the BM flock of ICAR-SRRC (CSWRI), Mannavanur, Kodaikanal, Tamil Nadu, India. The genetic trends were calculated by regression of average predicted breeding values using software WOMBAT for the traits BWT, 3WT, 6WT and GFY versus the animal’s birth year.

**Results:**

The least square means were 3.28±0.02 kg, 19.08±0.23 kg, 25.00±0.35 kg and 2.13±0.07 kg for BWT, 3WT, 6WT and GFY, respectively. Genetic trends were positive and highly significant (p<0.01) for BWT, while the values for 3WT, 6WT and GFY though positive, were not significant. The estimates of genetic trends in BWT, 3WT, 6WT and GFY were 5 g, 0.8 g, 7 g and 0.3 g/year gain and the fit of the regression shows 55%, 22%, 42% and 12% coefficient of determination with the regressed value, respectively. In this study, estimated mean predicted breeding value (kg) in BWT and 3WT, 6WT and GFY were 0.067, 0.008, 0.036 and −0.003, respectively.

**Conclusion:**

Estimates of genetic trends indicated that there was a positive genetic improvement in all studied traits and selection would be effective for the improvement of body weight traits and GFY of BM sheep.

## Introduction

During the last 40 years, extensive efforts have been made to improve the production potential of indigenous sheep through various research and developmental efforts by different agencies. The earlier efforts were aimed at improving sheep for fine wool production through introducing exotic fine wool inheritance or up-gradation using the indigenous improver wool or dual-purpose breeds on a limited scale. Though, considerable achievements have been made in developing new strains like Bharat Merino (BM). The flock of BM sheep has undergone 16 generations of multiple trait selection so far. The reliable estimates of genetic trends, i.e. the annual rate of the genetic change allow comparison of expected and realized genetic change in the experimental situations and assessment of progress in a particular trait.

A study of genetic trend in important economic traits is, therefore, necessary for knowing its performance status over the years to develop the breeding strategies for further genetic improvement of productivity of this flock of sheep. The estimation of genetic trends over time is problematic because of the difficulty in conducting experiments in uniform conditions over a period of several generations so that changes in performance of a selected population may reflect, in part, both environmental and genetic changes [1]. Theoretically, it is possible to simultaneously maintain a control population to remove the influence of environmental change [2], but this could prove expensive especially over a long period. Genetic response of growth rate may be enhanced by selecting lambs independently of their maternal effects [3,4]. Genetic trends for lamb performance traits in BM sheep have been estimated earlier with small data at semi-arid zone at Central Sheep and Wool Research Institute (CSWRI)Avikanagar, Rajasthan [5] when the flock of BM sheep had undergone seven generations in multiple trait selection.

The objective of this study was to estimate genetic trends for birth weight (BWT), 3WT, 6WT and GFY out of which the traits, 6WT and GFY were under selection in the BM sheep.

## Materials and Methods

### Ethical approval

All the experiments used in the study were carried out as per the approval from institutional animal ethics committee.

### Geographical location

Mannavanur is located at an altitude of 2030 m above MSL in the Western Ghats of southern India. The minimum and maximum ambient temperature ranges from 0°C to 5°C and 26°C to 30°C, respectively in this sub-temperate region, with the mean relative humidity between 15% and 90%. The rainfall is erratic and round the year with an annual mean rainfall of 1055 mm.

### Data and herd management

The data for the present study represents a total of 1652 BM lambs; progeny of 144 sires spread over 15 years starting from 2000 to 2014 was obtained from the BM flock of SRRC, Mannavanur. In addition to two different economic traits 6WT and GFY under selection, BWT and 3WT were used for analysis in BM genetic improvement project. The traits under study were BWT, weaning weight (3WT), 6 months weight (6WT) and grease fleece wool yield (GFY). A flock of around 400 BM sheep was maintained at the ICAR-SRRC (CSWRI), under semi-intensive with standard management system. The animals were allowed to graze from morning 8 am to evening 5 pm on the grazing area having grass species mostly kikuyu (*Pennisetumclandestinum*) having 11.35% crude protein (CP); 23.09% crude fiber (CF) and spear grass (*Heteropogoncontortus*) having 7.90% CP; 29.38% CF on dry matter basis. The ewes were housed in sheds during night. The animals were also provided concentrate mixture (total digestible nutrient 65% and digestible crude protein 12%) at 400 g/day/ewe during late pregnancy and lactation. In the remaining period, ewes were provided with concentrate mixture at 3000 g/day/ewe. Breeding was carried out in two seasons *viz*. spring breeding (March-April) and autumn breeding (September-October). The animals were shorn once in a year during March.

### Statistical analysis

Individual predicted breeding values were predicted for BWT, 3WT, 6WT and GFY. Breeding values were using an animal model. The mixed model analyses were performed using software WOMBAT [6] using an average information (Al) algorithm. Convergence was assumed when change of the value of the natural logarithm of the restricted likelihood function in two consecutive iterations was lower than 5 × 10^−4^. Single trait linear model used for analysis was

y= Xβ + Za +ε

Where, y is the vector of records; β, a, and ε are vectors of fixed, direct additive genetic and residual effects, respectively; association matrices X and Z. Assumptions in the model were 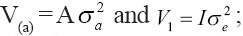 where, I is an identity matrix, A is the numerator relationship matrix between animals and 
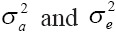
 are additive direct and residual variances, respectively.

The genetic trends were calculated by regression of average predicted breeding values versus the animal’s birth year.

## Results and Discussions

The least square means were 3.28±0.02 kg, 19.08±0.23 kg, 25.00±0.35 kg and 2.13±0.07 kg for BWT, 3WT, 6WT and GFY, respectively (Table-1). The least square mean of BWT of BM sheep was in close agreement with the results observed by Chopra *et al.*, Tomar *et al.*, Dixit *et al*. [7-9] as 3.31±0.01, 3.21±0.08, 3.1±0.03. However, they Chopra *et al.*, Tomar *et al.*, Dixit *et al*. [7-9] reported lower values of 15.67±0.07, 15.74±0.45, 15.0±0.2 kg body weight in 3 months of age and 22.39±0.10, 22.31±0.91, 21.6±0.2 kg body weight in 6 months of age in BM sheep reared under semiarid zone which could be due to the effect of scarce availability of grass and pasture on the traits considered in the present study. Similarly, Tomar *et al*. [10] also reported lower estimates of1.09±0.02 kg GFY in BM sheep under semiarid climatic zone of Rajasthan, when compared to the values of GFY in the present study. Different estimates could be due to differences in genetic merit of sires or could be due to differences in climate and management practices. The mean predicted breeding values (MPBV) for above traits per year has been tabulated in Table-1.

**Table-1:** MPBV of BM sheep in different years (mean difference).

Year of birth	BWT (kg) MPBV	Mean (kg)	3WT (kg) MPBV	Mean (kg)	6WT (kg) MPBV	Mean (kg)	GFY (kg) MPBV	Mean (kg)
2000	0.040	3.68	0.005	18.28	−0.002	26.11	−0.002	3.78
2001	0.027	3.70	0.009	19.88	−0.157	23.88	−0.003	3.14
2002	0.016	3.36	0.002	19.85	−0.253	22.93	−0.003	2.47
2003	0.064	3.31	0.008	16.61	−0.210	21.60	−0.001	1.93
2004	0.061	2.66	−0.015	17.34	0.203	25.24	−0.008	2.48
2005	0.072	3.30	0.011	18.14	−0.306	25.02	−0.005	2.40
2006	0.089	3.40	0.012	21.98	−0.233	28.04	−0.010	1.88
2007	0.084	3.70	0.015	20.01	−0.158	27.10	0.001	1.54
2008	0.043	3.23	0.009	18.17	−0.319	25.04	−0.003	1.77
2009	0.047	3.07	0.006	18.82	−0.069	23.01	0.000	1.88
2010	0.062	3.22	0.008	19.50	−0.024	25.97	−0.001	1.83
2011	0.069	3.29	0.012	20.82	0.056	24.88	−0.001	1.91
2012	0.099	3.18	0.013	20.19	−0.057	25.61	−0.002	1.67
2013	0.116	3.24	0.014	18.89	0.043	29.15	0.000	3.78
2014	0.116	3.16	0.013	18.28	−0.002	26.11	-	-

MPBV=Mean predicted breeding value, BM=Bharat merino, BWT=Birth weight, 3WT=Weaning weight, 6WT=6 months weight, GFY=Greasy fleece weight

### Genetic trends

The genetic trends express the outcome of a given genetic improvement program in a herd in terms of the rate of change in performance level of the herd a unit of time which is commonly referred to as “trends” of performance level of the herd. The evaluation of genetic trend gives an indication of genetic direction of the breed as well as the rate of genetic improvement from the time of application of the breeding program [11]. The estimates of genetic trends (g/year) for BWT, 3WT, 6WT and GFY traits were 5, 0.8, 7 and 0.3 and the fit of the regression shows 55%, 22%, 42% and 12% coefficient of determination with the regressed (R^2^) value, respectively (Table-2). Reason for lower (R^2^) value could be that these traits being quantitative, there are several environmental factors affecting them. The possible independent variables based on availability on data were included in the model. Estimated MPBV (kg) in BWT, 3WT, 6WT and GFY were 0.067, 0.008, 0.036 and −0.003, respectively. The magnitude of genetic trends estimated illustrate that there has been a highly significant and positive genetic improvement in BWT. However, the values for other traits though positive, were not significant.

**Table-2:** Estimates of genetic trends (Kg/year) for some of the growth traits in BM sheep.

Particulars	Least square Mean±SE (kg)	ΔG (kg/year)	Effect	R^2^
BWT	3.28±0.02	0.005+0.0268	**	0.55
Weaning weight	19.08±0.23	0.0008+0.0021	NS	0.22
6 months weight	25.00±0.35	0.0071x−0.021	NS	0.42
Grease fleece wool yield	2.13±0.07	0.0003x−0.0047	NS	0.12

** p<0.01.

ΔG=Genetic gain, R^2^=Regression fit for genetic trend, BM=Bharat merino, SE=Standard error

### BWT

**Figure-1:**
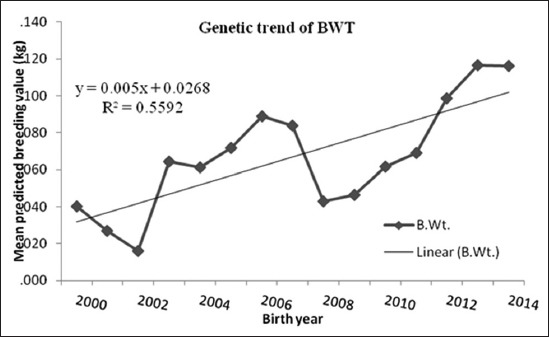
Mean of predicted breeding value of birth weight in each year of birth with genetic trend.

Means of predicted breeding values of BWT in each year of birth calculated from the four-trait analysis are shown in Figure-1. As perceived from the beginning of study period, ascendant but irregular genetic trend was noticed. However, the present genetic trends were positive and highly significant (p<0.01) for BWT, however, genetic trends attained of Arora *et al.*, Di *et al*. [12,13] for Malpura and Chinese superfine Merino sheep, respectively, were insignificant. The genetic trend value estimated for BWT in the current study (5 g/year) was similar value reported by earlier researchers in Dohne Merino in South Africa and Zandi Sheep in Khojir Sheep Breeding Station, located between Tehran and Abali [14,15]. However, the present value was higher the value reported by Dixit *et al*. [5] in BM sheep at semiarid zone at Rajasthan and in Arman sheep at Iran [3]. Furthermore, Barki sheep in RasElhekma research station of Egypt the estimated annual genetic gain was 15 g/year for BWT [16]. When compared to the present study, the value of genetic trend in Barki sheep [17], Kermani sheep[18] and Dormer breed at South African [19] was higher.

### Weaning weight

**Figure-2:**
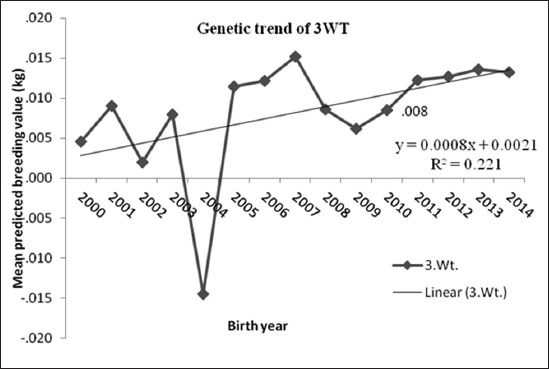
Mean of predicted breeding value of weaning weight in each year of birth with genetic trend.

Figure-2 shows the value of genetic trends during a period of 15-year for 3WT of BM sheep (0.8 g/year) was very lower than those of in Arman sheep (7 g/year) [3], in BM sheep (13 g/year) [5], in Ossimi (20 g/year) and Rahmani breeds (92 g/year) [1], and Ile de France breed (3.44g/year) [19]. Mokhtari and Rashidi [18] reported that mean of predicted breeding values for 3WT increased about 125 g/year in Kermani sheep and [15] Zandi sheep in Khojir Sheep breeding Station, located between Tehran and Abali (48 g/year). Furthermore, Lax *et al*. [20] reported mean increase for breeding value in 3WT was 620 g/year. The results of the above said three studies [15,18,20] were higher than that obtained in this study.

### 6 months weight

**Figure-3:**
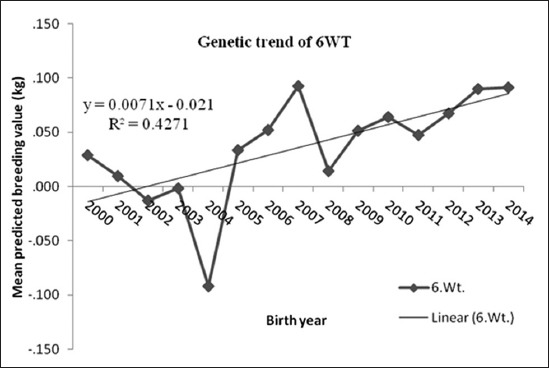
Mean of predicted breeding value of 6 months weight in each year of birth with genetic trend.

In Figure-3, mean of predicted breeding values of 6WT in each year of birth calculated from the four trait analysis are depicted. The current genetic trend estimate for 6WT indicated that increase a year as result of selection at genetic level was 7 g/year. The fit of the regression shows 42%coefficient of determination with the regressed value. This observation was in contrast to the earlier estimate of (−44 g/year) gain in BM sheep [5]. This estimate provides a good picture of the selection program for BM sheep as far as 6WT is concerned. Mokhtari and Rashidi [18] reported that there was a positive genetic trend for 6WT for Kermani sheep. Mohammadi *et al*. [21] reported that genetic trend was 21 g/year for 6WTin Zandi sheep. The results of the above said two studies [18,21] were higher than that obtained in this study. A higher estimate was observed in Rahmani sheep of Egypt (135 g/year) [1] and in Malpura sheep (61 g/year) [12].

### Greasy fleece wool yield

**Figure-4:**
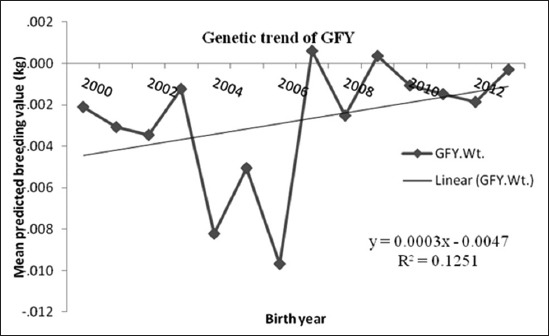
Mean of predicted breeding value of greasy fleece yield in each year of birth with genetic trend.

The genetic trend for GFY indicated that the genetic gain was 0.3 g/year, which was low, although in positive direction (Figure-4). But in the earlier studies [5], the estimate of genetic gain was 3 g/year in BM sheep. However, Arora *et al*. [12] reported similar estimate of genetic trends with a gain of 0.7 g/year in Malpura sheep. There is a need for reviewing the ongoing breeding programs for BM sheep. The genetic changes of GFY declined from 2002 to 2006, but for the other selected weights, plots of the MPBVs on year of birth indicated that there was an increase over time. The decrease of predicted breeding values mean in the year 2004-2006 was apparently due to selection of sires with low breeding value as well as poor management practice in the herd during that period.

## Conclusion

Usually, the genetic gain correspondents to the estimate of additive genetic variance of the breed and also the selection practices being followed. However, not only this but also the environmental determinants of variation have a significant impact on most of the production traits on which we have a very little control such as erratic rain fall, decrease in grazing area, and disease occurrence. There is a need to also unravel factors which increase the predictability of these variables for better management solutions. Estimates of genetic trends indicated that there was positive genetic improvement in all studied traits and selection would be effective for the improvement of body weight traits and GFYof BM sheep.

## Authors’ Contributions

PKM and SMKT designed the experiment. PKM, SRP, and GM conducted the experimental work and collected data. PKM, RV and SMKT were involved in analysis of the data. PKM, RP and GN were involved in scientific discussion, PKM, RP, GN and RV drafted and revised the manuscript. ASR monitored the overall work. All authors read and approved the final manuscript.
